# Internal fit of cobalt–chromium and zirconia implant abutments fabricated using different techniques: a micro–computed tomography study

**DOI:** 10.1186/s40729-026-00718-x

**Published:** 2026-10-13

**Authors:** Pedro Molinero-Mourelle, Seyed Ali Mosaddad, Rocio Cascos-Sanchez, Margarita Chevalier del Río, Gülce Çakmak, Andrea Roccuzzo, Miguel Gómez-Polo

**Affiliations:** 1 Department of Conservative Dentistry and Prosthodontics, Faculty of Dentistry, Complutense University of Madrid, Madrid, Spain; 2 Department of Reconstructive Dentistry and Gerodontology, School of Dental Medicine, University of Bern, Bern, Switzerland; 3 Complutense University of Madrid, Ramon y Cajal Research Institute (IRYCIS), Madrid, Spain; 4 Department of Research Analytics, Saveetha Institute of Medical and Technical Sciences, Saveetha Dental College and Hospitals, Saveetha University, Chennai, India; 5 Departament of Radiology, Rehabilitation and Physiotherapy, Faculty of Medicine, Complutense University of Madrid, Madrid, Spain; 6 Department of Prosthodontics, Geriatric Dentistry and Craniomandibular Disorders, Corporate Member of Freie Universität Berlin and Humboldt- Universität zu Berlin, Charité-Universitätsmedizin Berlin, Berlin, Germany; 7 Department of Prosthodontics, Faculty of Dentistry, Biruni University, Istanbul, Turkey; 8 Shanghai Perio-Implant Innovation Center, Institute of Integrated Oral, Craniofacial and Sensory Research, Shanghai Ninth People’s Hospital, College of Stomatology, National Center of Stomatology, National Clinical Research Center for Oral Diseases, Shanghai Key Laboratory of Stomatology, Shanghai Jiao Tong University School of Medicine, Shanghai Jiao Tong University, Shanghai, China; 9 Department of Periodontology, School of Dental Medicine, University of Bern, Bern, Switzerland

**Keywords:** Dental implants, Dental implant-abutment design, Tomography, X-ray computed, Dental prosthesis design, Dental materials

## Abstract

**Purpose:**

To evaluate the internal fit at the implant-abutment interface of zirconia and cobalt–chromium abutments fabricated using different techniques by micro-computed tomography (micro-CT).

**Methods:**

Twenty screw-retained abutments were divided into 4 groups (*n* = 5): milled zirconia (ZrCAD), milled cobalt–chromium (Co-CrMill), laser-sintered cobalt–chromium (Co-CrLS), and cast cobalt–chromium (Co-CrCL). Specimens were subjected to cyclic loading (300,000 cycles, 200 N) and thermocycling (10000 cycles, 5 to 50 °C). Internal fit was assessed by micro-CT at 3 regions and 3 spatial planes (180 measurements). Data were analyzed with Kruskal–Wallis and Bonferroni-Holm tests (α = 0.05).

**Results:**

Significant differences in internal fit were found (*p* < 0.05). Co-CrLS showed the highest internal gap values (26.75 ± 7.05 μm, 87.00 ± 3.26 μm, 86.50 ± 5.76 μm). Significant differences were observed between Co-CrLS and ZrCAD (Region 1, *p* = 0.003), Co-CrMill (Regions 1 and 2, *p* = 0.003–0.004), and Co-CrCL (Regions 1 and 3, *p* = 0.003–0.001). Total values also differed between Co-CrMill and Co-CrLS (*p* = 0.012) and Co-CrCL and Co-CrLS (*p* = 0.002).

**Conclusions:**

Internal fit differed among the evaluated workflows. Laser-sintered cobalt–chromium abutments showed the least favorable adaptation, whereas milled and cast cobalt–chromium showed lower gap values; zirconia showed intermediate descriptive values. The clinical significance of these differences remains uncertain.

**Clinical trial number:**

Not applicable.

## Background

Single implant-supported restorations are a predictable and widely used treatment option for the rehabilitation of partially edentulous patients, with high medium- and long-term survival rates having been consistently reported [1–3]. Nevertheless, these restorations are not free from technical and biological complications, and the precision of the implant–abutment interface remains a critical factor for their long-term performance [4–6]. Inadequate fit at this interface has been associated with mechanical complications, such as screw loosening, abutment instability, component fracture, and unfavorable stress concentrations, as well as biological complications, including microbial colonization and peri-implant tissue breakdown [7–10].

The quality of the implant-abutment fit is influenced by several factors, including connection design, material properties, manufacturing accuracy, and the use of original or compatible components [7, 8]. The enhanced sealing and mechanical stability of conical implant–abutment connections are primarily attributed to frictional engagement generated by relative axial displacement between the mating tapered surfaces during abutment seating. This wedging effect produces interfacial contact pressure that limits micromovement and microgap formation at the connection [9, 11]. In parallel, the development of computer-aided design and computer-aided manufacturing (CAD-CAM) technologies has enabled the fabrication of customized prosthetic components with greater standardization and potentially improved precision compared with conventional workflows [8, 12]. Despite these advances, discrepancies at the implant-abutment interface continue to be reported, indicating that prosthetic material and fabrication technique remain relevant determinants of fit [7, 8, 13].

Cobalt–chromium (Co-Cr) and zirconia are established materials for implant-supported prosthetic components [14–17]. Studies have documented the established use of Co-Cr alloys in fixed implant-supported prosthodontics [18–20]. Their selection is supported by high strength and elastic modulus, resistance to wear and corrosion, and lower cost relative to noble-metal alloys [20, 21]. Zirconia has gained popularity because of its biocompatibility and esthetic advantages, particularly in situations with limited soft-tissue thickness [14, 16, 17]. In addition to material selection, the fabrication technique may substantially affect the accuracy of the definitive prosthetic component. Co-Cr restorations can be produced by conventional casting, milling, or laser sintering, and each technique involves distinct processing steps that may influence dimensional accuracy and, consequently, the quality of the implant-abutment fit [13, 15, 22, 23].

The assessment of implant-abutment fit has traditionally relied on optical microscopy and scanning electron microscopy (SEM), which have mainly been used to evaluate marginal adaptation or external discrepancies [4, 7, 8, 15, 23, 24]. These methods have provided valuable information, but they are limited by their two-dimensional nature and, when internal regions are investigated, often require destructive specimen sectioning [7, 8]. Likewise, functional assessments such as microleakage analyses have shown that imperfections at the interface may permit fluid and bacterial penetration, reinforcing the clinical relevance of fit quality [10, 25]. However, neither external measurements nor leakage tests fully characterize the three-dimensional geometry of the implant-abutment interface [7, 25].

Micro-computed tomography (micro-CT) has become a powerful non-destructive imaging modality that enables high-resolution three-dimensional evaluation of internal structures [26–28]. In implant prosthodontics, this technique allows visualization of the internal implant-abutment interface and assessment of fit at different regions without damaging the specimens [26, 27]. In addition to preserving the samples, micro-CT enables analysis of the spatial distribution of internal discrepancies across multiple planes, providing information that cannot be obtained with conventional sectioning-based methods [26, 27, 29]. Although micro-CT has increasingly been used in dental research, studies specifically evaluating the internal fit of implant-supported abutments fabricated from different materials and by different manufacturing techniques remain limited [26, 27, 30].

Therefore, the aim of the present in vitro study was to assess the internal fit at the implant-abutment interface by using micro-computed tomography, comparing milled Co-Cr, laser-sintered Co-Cr, cast Co-Cr, and milled zirconia abutments at three different regions of a conical connection implant system. The null hypothesis was that no significant differences in internal fit would be found among the evaluated materials and fabrication techniques.

## Methods

In this comparative in vitro study, twenty screw-retained implant-supported abutments were evaluated and divided into four groups based on material and fabrication technique (*n* = 5 per group).

The sample size was calculated using a statistical software program (nQuery Advisor, version 8.5.2; Statistical Solutions Ltd., Cork, Ireland). For a 2-sided comparison of 2 independent groups, the calculation assumed an expected between-group mean difference of 5.0 μm and a common within-group standard deviation of 2.5 μm, corresponding to a standardized effect size of 2.0. With a significance level of 0.05 and statistical power of 70%, the required sample size was 5 specimens per group. A power of 70% was considered acceptable because of the exploratory pilot design of the study.

### Specimen preparation

Twenty osteotomies were prepared in polymethylmethacrylate resin blocks using a parallelizing drill press to ensure standardized implant positioning and angulation (Mechanical Workshop, Faculty of Physics, Complutense University of Madrid). The resin blocks had an elastic modulus of 3000 MPa, approximating cancellous bone properties [31]. One conical connection implant (4.1 × 8.5 mm; Ocean, Avinent Implant System, Santpedor, Barcelona, Spain; reference no. 1590) was placed in each block according to the manufacturer’s instructions.

Twenty identical screw-retained implant-supported one-piece restorations were designed based on a mandibular first premolar morphology. The restorations were fabricated in 2 materials: milled 3 mol% yttria-stabilized tetragonal zirconia polycrystal doped with alumina (3Y-TZP-LA) and cobalt-chromium (Co-Cr), the latter produced using different fabrication techniques. The digital workflow was performed by scanning an extraoral scan body (Core 3D Centers, Dental Direkt GmbH, Spenge, Germany) with a laboratory desktop scanner (Biomet Zfx, Zimmer ZFX GmbH, Dachau, Germany). A standard tessellation language file was generated and imported into a CAD software program (Exocad GmbH, Darmstadt, Germany) to standardize the prosthetic design.

### Abutment fabrication

Specimens were allocated to four groups according to material and fabrication technique. The zirconia group consisted of milled zirconia abutments, whereas the Co-Cr groups included milled, laser-sintered, and cast abutments.

Zirconia abutments (ZrCAD) were fabricated using original implant components and yttria-stabilized tetragonal zirconia, according to International Organization for Standardization (ISO 13356:2015) [32], on a 5-axis precision milling machine (Roland DWX-52DC; Roland DG Deutschland GmbH, Willich, Germany).

Co-Cr abutments were fabricated from alloys that comply with the International Organization for Standardization (ISO 5832-12:2019) [33]. The milled Co-Cr abutments (Co-CrMill) were produced in the same processing center by using a 5-axis milling machine (DMG Sauer HSC 20 Linear; DMG MORI AKTIENGESELLSCHAFT, Bielefeld, Germany). The laser-sintered Co-Cr abutments (Co-CrLS) were fabricated using a selective laser melting (SLM) system (SLM 125; SLM Solutions Group AG, Lübeck, Germany) from the same digital design. The cast Co-Cr (Co-CrCL) abutments were fabricated using the lost-wax technique. A previously milled framework was embedded in a heavy-body addition silicone (Platinum 85 TOUCH; Zhermack SpA, Rome, Italy) to obtain wax patterns, which were subsequently transferred to a plastic castable abutment (Dental Smart Solutions, Terrats Medical S.L., Barberá del Vallès, Spain) and cast by induction heating and centrifugal casting in ambient atmosphere. The material compositions and manufacturers are summarized in Table 1.

**Table 1:** Materials and fabrication groups evaluated in the study

Group	Material composition	Material manufacturer	Prosthetic manufacturer
ZrCAD	3 mol% yttria-stabilized tetragonal zirconia polycrystal (3Y-TZP-A)	Dental Direkt GmbH, Spenge, Germany	CORE 3D PROTECH, S.L.U., Santpedor, Spain
Co-CrMill	63% Co, 29% Cr, 6% Mo, < 1% Nb, Si, Mn, Fe	Dental Direkt GmbH, Spenge, Germany	CORE 3D PROTECH, S.L.U., Santpedor, Spain
Co-CrLS	59% Co, 25% Cr, 9.5% W, 3.5% Mo, 1% Si	S & S Scheftner GmbH – Dental Alloys, Mainz, Germany	Prótesis S.A., Madrid, Spain
Co-CrCL	61% Co, 24% Cr, 8% W, 2.5% Mo, 1% Nb, 1% Mn, 1% Si, 1% Fe	Dentalforschung Schleicher GmbH, Riedenburg, Germany	Riosa Laboratory, Pozuelo de Alarcón, Spain

3Y-TZP-A, yttria-stabilized tetragonal zirconia polycrystal doped with alumina; Co, cobalt; Co-CrCL, cast cobalt–chromium abutments; Co-CrLS, laser-sintered cobalt–chromium abutments; Co-CrMill, milled cobalt–chromium abutments; Cr, chromium; Fe, iron; Mn, manganese; Mo, molybdenum; Nb, niobium; Si, silicon; W, tungsten; ZrCAD, CAD-CAM milled zirconia abutments

### Mechanical aging

All abutments were tightened to their respective implants to 35 Ncm, according to the manufacturer’s recommendations. Specimens were subjected to cyclic loading using a chewing simulation machine (Instron; Euroortodoncia S.A. and Complutense University of Madrid; Zwick/Roell testXpert II software). A total of 300,000 loading cycles were applied at a force of 200 N, with loading intervals of 0.5 s, at an angle of 30° relative to the implant’s long axis [34]. The load was applied to the occlusal surface of the restorations using a 2-mm polytetrafluoroethylene cylinder.

After cyclic loading, thermocycling was performed in a thermocycling device (Complutense University of Madrid, Spain) in artificial saliva for 10,000 cycles between 5 °C and 50 °C, with a dwell time of 55 s.

Following mechanical aging, all specimens were visually inspected for fractures, deformation, or other complications by using magnification loupes (×2.5; ExamVision ApS, Samsø, Denmark), and a tactile mobility test was performed with dental pliers. The specimens were then cleaned using a polishing system for metal and ceramic (Komet Dental; Gebr. Brasseler GmbH & Co. KG, Lemgo, Germany) and stored in an airtight container filled with distilled water for 24 h before micro-computed tomography analysis.

### Micro-computed tomography analysis

Internal fit was assessed using a micro-computed tomography system consisting of a compact pulse microfocus X-ray source and a flat-panel complementary metal–oxide–semiconductor detector. Three-dimensional reconstructions of the implant–abutment assemblies were obtained using a reconstruction software program (Octopus Reconstruction, version 8.9; TESCAN Orsay Holding, a.s., Brno, Czech Republic) [35].

**Fig. 1:**
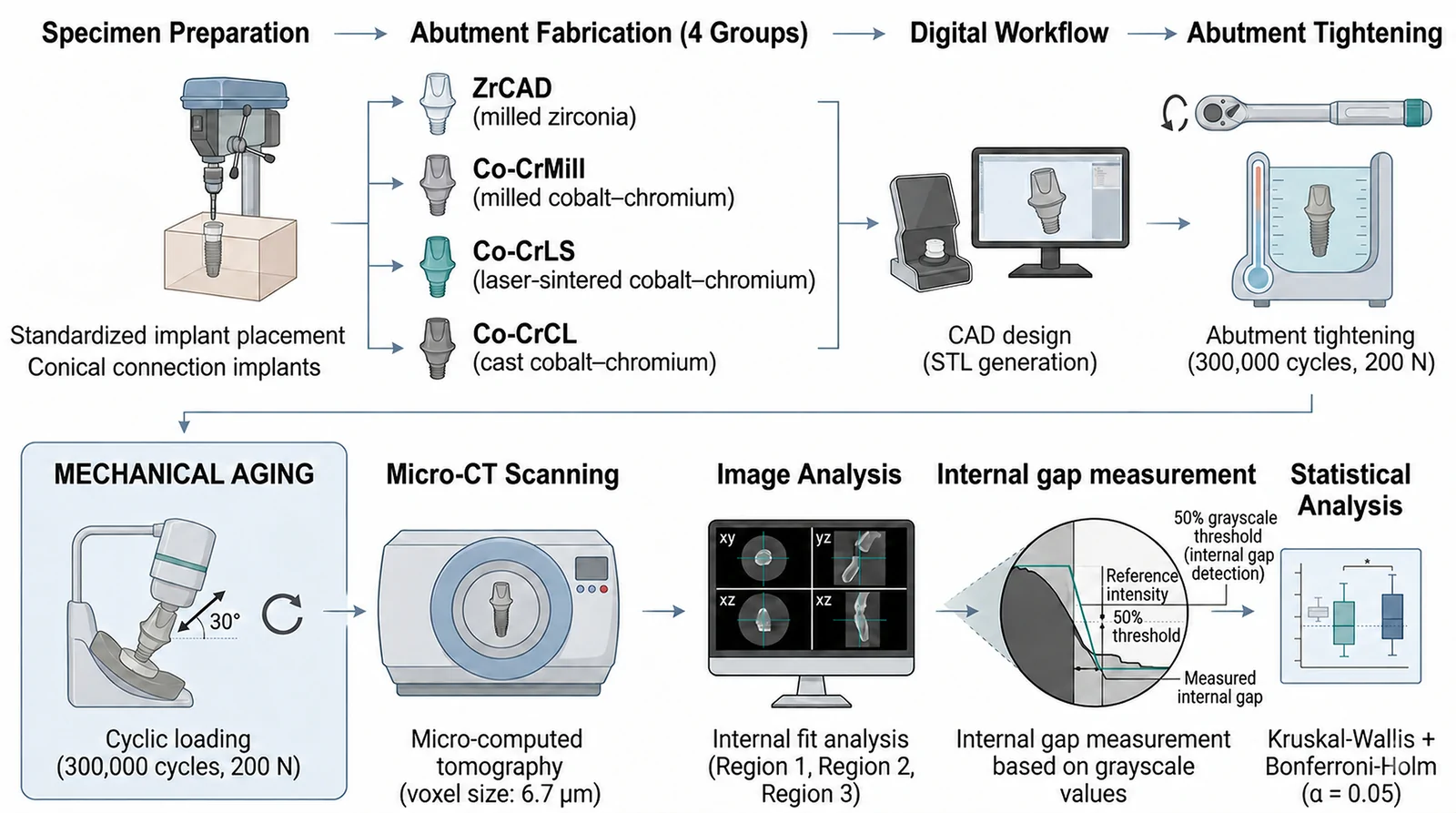
Schematic representation of the experimental workflow, including specimen preparation, abutment fabrication, mechanical aging, micro-computed tomography (micro-CT) analysis, and internal fit measurement

The source-to-detector distance was set at 790 mm and the source-to-object distance at 105.33 mm, resulting in a magnification factor of 7.5 and a voxel size of 6.7 μm. The scanning parameters were 85 kV and 150 µA. A total of 1000 projections were acquired over a 360° rotation range in analog integration mode with 2 integrations to reduce image noise (Fig. 1).

Image reconstruction was performed using a filtered back-projection algorithm implemented in the reconstruction software [36]. Projection images were normalized by selecting a region free of object shadows and using its mean pixel value as a reference. Ring artifact correction and spot removal procedures were applied to minimize detector-related artifacts.

**Fig. 2:**
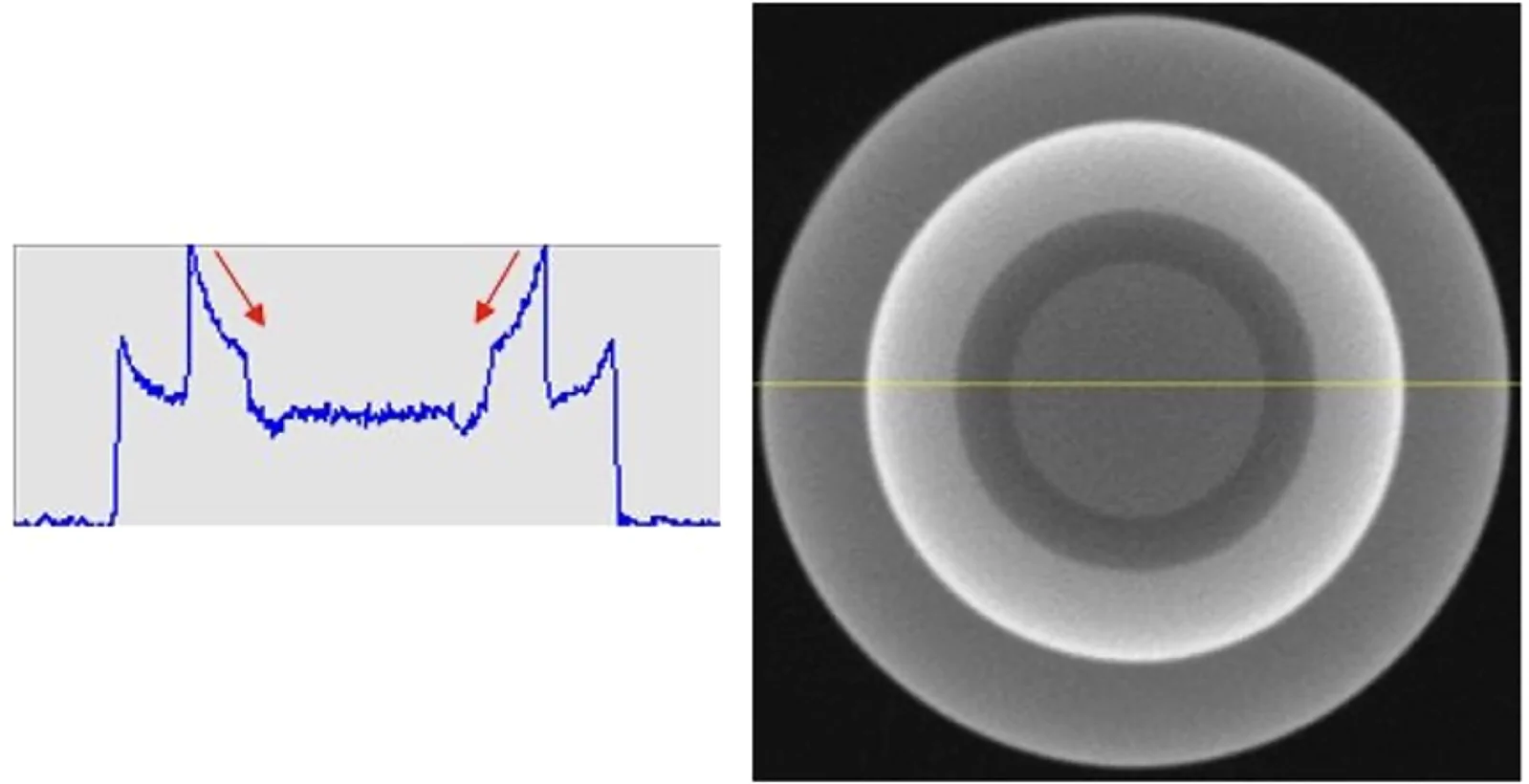
Axial micro-computed tomography slice of the implant-abutment interface showing beam-hardening artifacts

To reduce beam-hardening artifacts (Fig. 2) caused by high-density materials, two corrective approaches were used. First, beam pre-hardening was performed by placing a 3-mm filter at the X-ray tube exit. Second, a polynomial beam-hardening correction was applied during reconstruction by converting normalized attenuation values to their negative logarithms and applying a polynomial correction. The maximum spatial resolution achieved with this system was 6.7 μm.

### Internal fit assessment

**Fig. 3:**
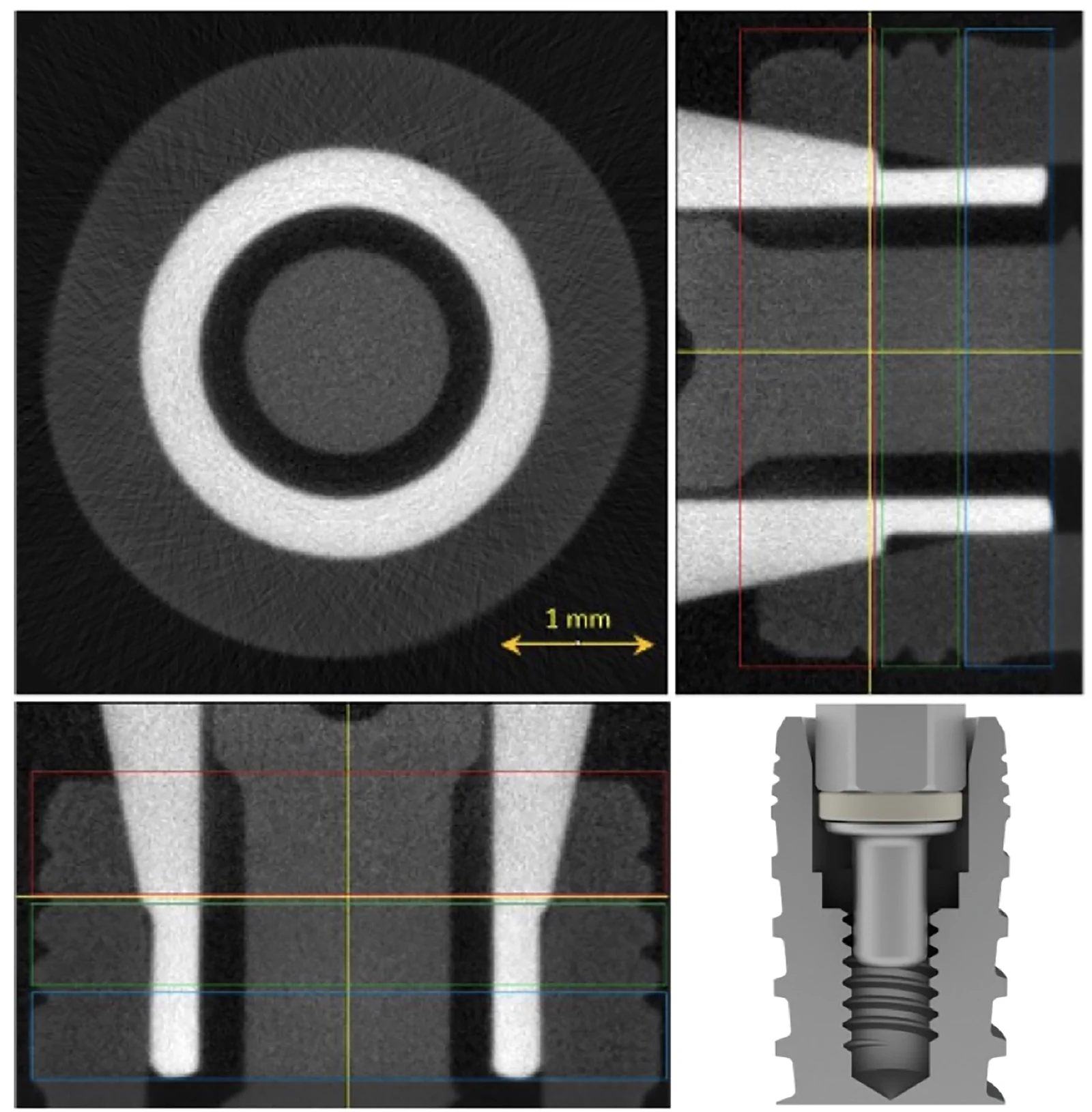
Micro-computed tomography image illustrating the evaluated regions for internal fit assessment at the implant-abutment interface

Volume visualization and image analysis were performed using an image analysis software program (Fiji; National Institutes of Health, Bethesda, MD, USA) [37]. The reconstructed volumes were analyzed in the three orthogonal planes (xy, yz, and xz) (Fig. 3). Internal fit was evaluated in three predefined regions of the implant–abutment interface, corresponding to the upper, middle, and lower zones.

**Fig. 4:**
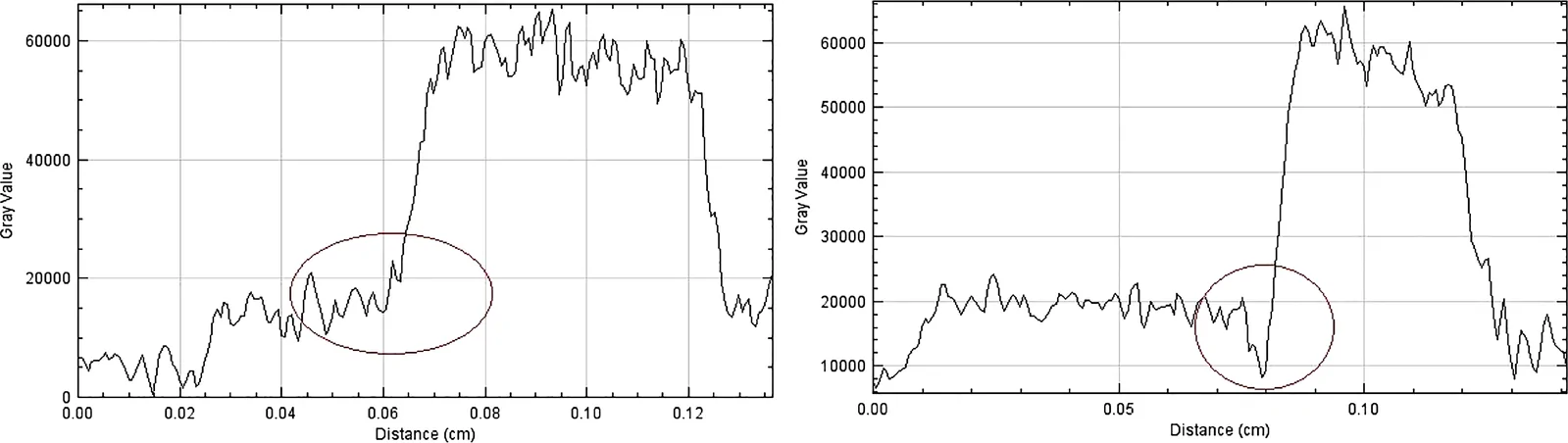
Representative micro-computed tomography images showing the presence and absence of an internal gap at the implant-abutment interface. Left: absence of detectable internal gap; right: presence of internal gap

**Fig. 5:**
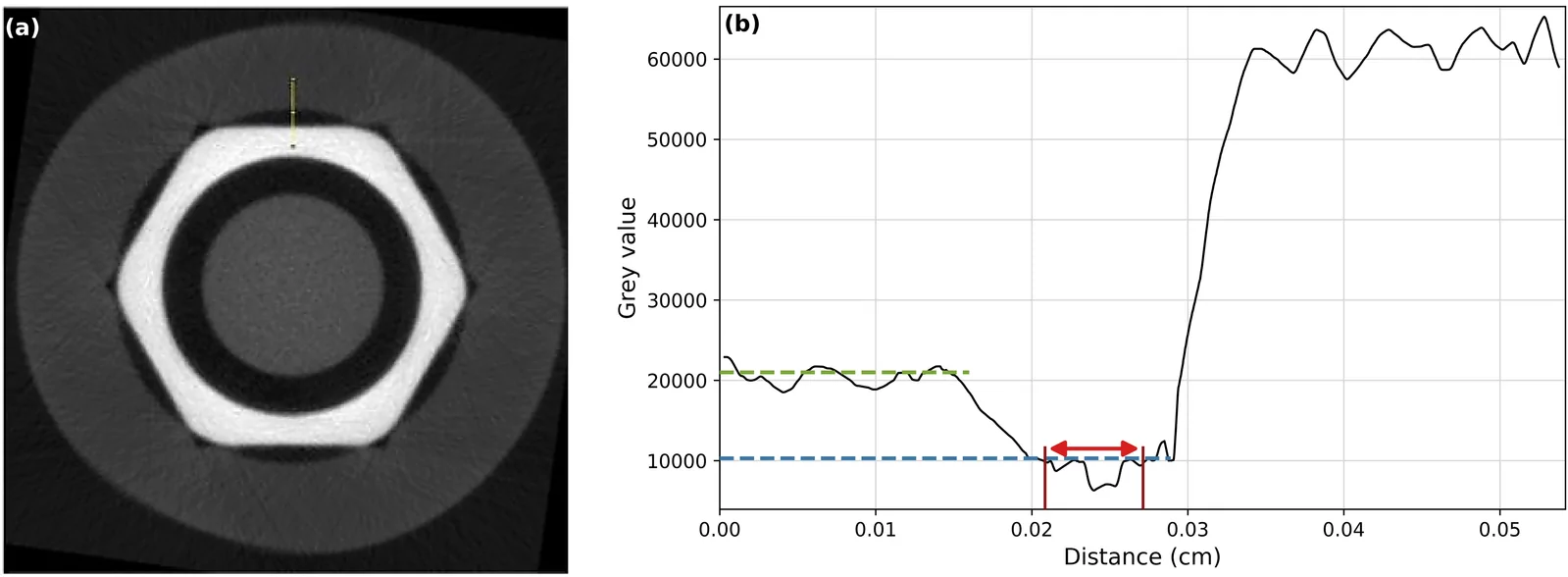
Measurement of internal gap width based on grayscale pixel values along the selected profile line. In the right image, the mean pixel value of the reference area (green line) was 2100. Internal gaps were identified when the pixel value decreased to 1050 (50% reduction, blue line), and gap width was measured as the distance between the two corresponding points (red lines)

For each specimen, the interface was examined in slices parallel to the three spatial planes, and gray-value profiles were obtained across the implant–abutment junction. Internal gaps were identified when a 50% decrease in the mean pixel value was observed at the interface (Fig. 4). Gap width was defined as the linear distance between the point at which the pixel value decreased by 50% and the point at which it recovered within the connection area (Fig. 5).

Nine measurements were obtained per specimen, corresponding to the evaluation of the three regions in the three spatial planes, yielding 45 measurements per group and a total of 180 for analysis.

### Statistical analysis

Statistical analysis was performed using a statistical software program (IBM SPSS Statistics, version 25.0; IBM Corp., Armonk, NY, USA). Descriptive statistics, including mean values and standard deviations, were calculated for internal fit measurements. The normality of the data distribution was assessed using the Shapiro–Wilk test. Because the data were not normally distributed, intergroup comparisons were performed using the Kruskal–Wallis test, followed by Bonferroni–Holm correction for multiple comparisons. The level of significance was set at α = 0.05.

## Results

**Fig. 6:**
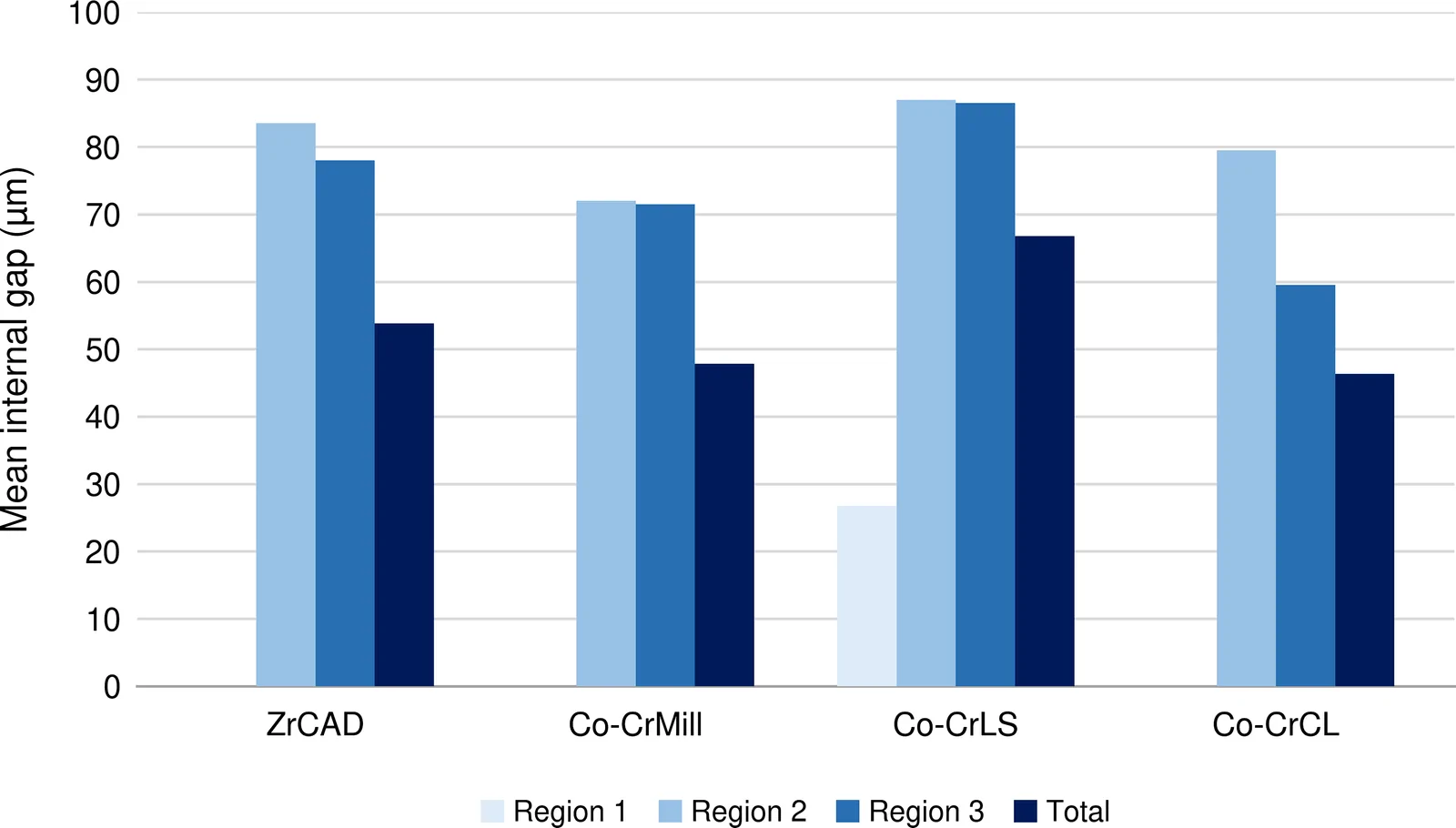
Mean internal gap values (µm) according to material and fabrication technique at the different regions of the implant–abutment interface. Region 1, upper region; Region 2, middle region; Region 3, lower region. ZrCAD, CAD-CAM milled zirconia abutments; Co-CrMill, milled cobalt–chromium abutments; Co-CrLS, laser-sintered cobalt–chromium abutments; Co-CrCL, cast cobalt–chromium abutments

A total of 180 measurements were obtained, corresponding to 45 measurements per group. Mean internal gap values and standard deviations are presented in Table 2 and illustrated in Fig. 6, while pairwise comparisons are summarized in Table 3.

**Table 2:** Descriptive micro-computed tomography analysis of internal fit (mean ± standard deviation, µm) at the three evaluated regions

Group	n	Region 1 (mean ± SD)	Region 2 (mean ± SD)	Region 3 (mean ± SD)	Total (mean ± SD)
ZrCAD	5	0.00 ± 0.00	83.50 ± 5.18	78.00 ± 3.71	53.83 ± 2.25
Co-CrMill	5	0.00 ± 0.00	72.00 ± 5.42	71.50 ± 5.18	47.83 ± 3.47
Co-CrLS	5	26.75 ± 7.05	87.00 ± 3.26	86.50 ± 5.76	66.75 ± 2.33
Co-CrCL	5	0.00 ± 0.00	79.50 ± 4.11	59.50 ± 2.09	46.33 ± 1.92

Co-CrCL, cast cobalt–chromium abutments; Co-CrLS, laser-sintered cobalt–chromium abutments; Co-CrMill, milled cobalt–chromium abutments; n, number of specimens; SD, standard deviation; ZrCAD, CAD-CAM milled zirconia abutments

**Table 3:** Pairwise comparisons of internal fit according to material and fabrication technique at the evaluated regions

Comparison	Region	Mean difference	SE	Test statistic	Sig.1	Sig.2
ZrCAD vs. Co-CrMill	1	0.000	2.845	0.000	1.000	1.000
	2	-8.600	3.701	-2.324	0.020	0.121
	3	-4.100	3.723	-1.101	0.271	1.000
	T	-6.300	3.735	-1.687	0.092	0.550
ZrCAD vs. Co-CrCL	1	0.000	2.845	0.000	1.000	1.000
	2	-3.300	3.701	-0.892	0.373	1.000
	3	-9.900	3.723	-2.659	0.008	0.047
	T	-8.100	3.735	-2.169	0.030	0.181
ZrCAD vs. Co-CrLS	1	-10.000	2.845	-3.515	0.000	0.003
	2	-3.900	3.701	-1.054	0.292	1.000
	3	-4.400	3.723	-1.182	0.237	1.000
	T	-5.200	3.735	-1.392	0.164	0.983
Co-CrMill vs. Co-CrCL	1	0.000	2.845	0.000	1.000	1.000
	2	-5.300	3.701	-1.432	0.152	0.913
	3	-5.800	3.723	-1.558	0.119	0.716
	T	-1.800	3.735	-0.482	0.630	1.000
Co-CrMill vs. Co-CrLS	1	-10.000	2.845	-3.515	0.000	0.003
	2	-12.500	3.701	-3.378	0.001	0.004
	3	-8.500	3.723	-2.283	0.022	0.135
	T	-11.500	3.735	-3.079	0.002	0.012
Co-CrCL vs. Co-CrLS	1	10.000	2.845	3.515	0.000	0.003
	2	7.200	3.701	1.946	0.052	0.310
	3	14.300	3.723	3.841	0.000	0.001
	T	13.300	3.735	3.561	0.000	0.002

Co-CrCL, cast cobalt–chromium abutments; Co-CrLS, laser-sintered cobalt–chromium abutments; Co-CrMill, milled cobalt–chromium abutments; SE, standard error; T, total; ZrCAD, CAD-CAM milled zirconia abutments

Statistical analysis revealed significant differences among groups across specific regions and in overall values (*p* < 0.05). Pairwise comparisons with Bonferroni-Holm correction showed statistically significant differences between ZrCAD and Co-CrCL at region 3, between ZrCAD and Co-CrLS at Region 1, between Co-CrMill and Co-CrLS at Regions 1 and 2, and between Co-CrCL and Co-CrLS at Regions 1 and 3. In addition, statistically significant differences in the total internal gap values were observed between Co-CrMill and Co-CrLS and between Co-CrCL and Co-CrLS (*p* < 0.05).

Among all groups, Co-CrLS demonstrated the least favorable internal fit, with the highest mean internal gap values at all 3 regions: 26.75 ± 7.05 μm at region 1, 87.00 ± 3.26 μm at region 2, and 86.50 ± 5.76 μm at region 3. At Region 1, Co-CrLS showed significantly greater internal gap values than ZrCAD, Co-CrMill, and Co-CrCL (*p* < 0.05). At Region 2, Co-CrLS also showed significantly greater internal gap values than Co-CrMill (*p* < 0.05).

At Region 3, Co-CrCL exhibited the most favorable internal fit, with the lowest mean internal gap value (59.50 ± 2.09 μm). This value was significantly lower than those observed for Co-CrLS and ZrCAD (*p* < 0.05).

A consistent regional pattern was observed, with lower internal gap values in the upper region and comparatively higher values in the middle and lower regions across most groups.

## Discussion

The present in vitro study evaluated the internal fit of zirconia and cobalt-chromium implant abutments fabricated using different techniques using micro-computed tomography. The findings showed differences in internal fit among the evaluated groups; however, the independent effects of material and fabrication technique could not be distinguished because zirconia was evaluated only with milling, whereas cobalt–chromium was evaluated with milling, laser sintering, and casting. Co-CrLS showed the least favorable internal fit, with the highest internal gap values at all evaluated regions, whereas Co-CrMill and Co-CrCL demonstrated the most favorable overall values. In addition, descriptively, lower internal gap values were observed in the upper region of the implant-abutment interface, whereas higher values were generally found in the middle and lower regions. Accordingly, the null hypothesis that no differences in internal fit would be found among the evaluated groups was rejected.

These findings suggest that differences among the evaluated manufacturing workflows may influence implant–abutment adaptation, even when the same implant system, restorative design, and aging protocol are used. This agrees with previous reports showing that misfit may originate during scanning, digital design, machining, additive manufacturing, or conventional laboratory procedures, thereby affecting the final adaptation of implant-supported components [7, 8, 13]. Although CAD-CAM workflows have improved standardization and reproducibility, dimensional inaccuracies may still occur depending on the manufacturing route. In the present study, the unfavorable performance of Co-CrLS may be related to the intrinsic characteristics of layer-by-layer fabrication, including thermal shrinkage, residual stresses, and local distortion during melting and postprocessing. This interpretation is supported by previous studies in which the fabrication method significantly affected adaptation, and laser-based fabrication did not necessarily produce the most favorable fit values [4, 30].

A critical aspect of these findings is their relationship with previous SEM-based analyses conducted under identical experimental conditions [15]. In that study, Co-CrMill exhibited the smallest marginal microgap, followed by ZrCAD, whereas Co-CrLS and Co-CrCL showed the largest external marginal discrepancies [15]. In the present study, Co-CrLS also revealed the least favorable behavior; however, Co-CrCL presented comparatively favorable overall internal values, and the ranking of the groups was not identical to that observed with SEM. This should not be interpreted as a contradiction, but rather as the consequence of the different variables evaluated by both methods. The SEM study assessed external marginal microgaps at selected peripheral points, whereas the present study obtained discrete linear internal-gap measurements from reconstructed micro-CT volumes at three predefined regions in three orthogonal planes. Although this approach allowed nondestructive sampling of internal portions of the connection, it did not provide a comprehensive three-dimensional characterization of the interface. Therefore, external marginal adaptation and the sampled internal gap measurements represent different aspects of the implant–abutment interface and should not be assumed to show identical patterns. A component may show less favorable marginal adaptation while exhibiting comparatively better seating at the sampled internal locations, or vice versa [9, 15, 38]. Comprehensive three-dimensional characterization would require volumetric or surface-based outcomes, such as total interfacial gap volume, the proportion of contacting surfaces, or spatial gap-distribution mapping [9, 39, 40].

This distinction is particularly important for the Co-CrCL group. In the SEM study, the cast abutments demonstrated unfavorable marginal microgap values, whereas in the present micro-CT analysis they showed comparatively favorable overall internal values [15]. A plausible explanation is that conventional casting may induce distortions or irregularities that are more evident at the marginal portion of the interface, while the deeper regions of the connection may still achieve relatively acceptable seating. In addition, SEM and micro-CT are based on different measurement principles. SEM directly measures the visible external gap at high magnification, whereas micro-CT assesses internal fit indirectly from grayscale transitions within reconstructed volumes and is influenced by voxel size, reconstruction algorithms, and artifact correction [9, 38]. Consequently, both techniques should be regarded as complementary rather than interchangeable methods for evaluating the implant-abutment interface.

The findings should also be interpreted together with the previously published microleakage study performed under the same experimental conditions [25]. In that investigation, the CAD-CAM groups generally revealed more favorable sealing behavior, whereas Co-CrLS and Co-CrCL presented higher bacterial leakage, with Co-CrLS showing the highest bacterial counts [25]. The fact that Co-CrLS showed the least favorable internal fit in the present study is in agreement with those findings and supports the concept that poor adaptation may contribute to bacterial penetration. However, the relationship between internal fit and leakage does not appear to be strictly linear. Although Co-CrCL exhibited relatively favorable overall internal values in the present study, it showed unfavorable microleakage behavior in the previous investigation [25]. This suggests that bacterial penetration may depend not only on the magnitude of the internal gap, but also on the continuity and location of the defect, the geometry of the leakage pathway, the adaptation of the marginal region, and the behavior of the interface under cyclic loading and thermal stress [25, 41].

Regarding the current literature, previous micro-CT studies have generally reported lower gap values for conical connection implants than for non-conical or internal hexagon configurations [26, 42]. Scarano et al. found nearly complete adaptation in cone Morse connections, whereas larger gaps were observed in internal hexagon, screwed, or trilobed configurations [26, 42]. He et al. further demonstrated that connection design and oblique loading influence contact behavior and microgap formation at the implant-abutment interface [9]. More recently, Ziada and Beleidy reported that connection design and abutment material both affected internal fit, and that zirconia tended to show greater gaps than metallic abutments, although all values remained within clinically acceptable ranges [28]. Likewise, Díaz et al. found that original milled titanium abutments demonstrated lower vertical misfit than laser-sintered Co-Cr abutments, and that connection type significantly affected misfit behavior [4]. Similar findings were reported by Gonzalo et al., who observed that milled titanium abutments showed lower marginal misfit values than laser-sintered Co-Cr abutments in internal implant-abutment connections, reinforcing the concept that additive manufacturing of Co-Cr components may be associated with less favorable adaptation than subtractive manufacturing approaches [23]. In contrast, Zuskova et al., in a study on CAD-CAM crown copings, reported no significant difference in overall fit between milled and laser-sintered frameworks [43]. This apparent discrepancy suggests that the influence of manufacturing technique on fit may depend heavily on the type of restoration, the geometry of the interface being assessed, and the measurement method employed.

Compared with those reports, the internal gap values observed in the present study were higher. Several methodological differences may explain this. First, this study included zirconia and cobalt-chromium abutments fabricated by milling, laser sintering, and casting, rather than stock titanium components only [23, 44]. Second, all specimens were subjected to cyclic loading and thermocycling before evaluation, which may have altered the seating condition of the components. Third, customized abutments were evaluated, for which manufacturing variability may be greater than for prefabricated components. Therefore, the higher values observed in the present study are likely related to both material- and technique-dependent factors and to the more clinically challenging aging protocol used.

Another point that requires cautious interpretation is the clinical meaning of the measured values. Historically, acceptable microgap ranges have often been proposed based on external or marginal assessments, with wide ranges reported in the literature [7, 8]. However, these thresholds were not established for discrete linear internal-gap measurements derived from micro-CT datasets. Accordingly, it would be inappropriate to classify the present values as clinically acceptable or unacceptable solely on the basis of thresholds derived from external microscopic analyses. Under these circumstances, the present findings are more informative as a comparative assessment of the influence of different fabrication techniques than as an attempt to define an absolute clinical cutoff for internal fit.

From a clinical standpoint, the results suggest that the choice of manufacturing technique may affect the quality of implant-abutment adaptation and, potentially, the long-term mechanical and biological behavior of implant-supported restorations. Under the conditions of this study, Co-CrMill exhibited favorable internal fit, whereas Co-CrLS showed the least favorable values, suggesting that subtractive manufacturing may provide more stable connection adaptation than laser sintering when precision at the implant-abutment interface is critical. At the same time, the differences observed between the present micro-CT results and the previous SEM findings indicate that a single evaluation method is insufficient to fully characterize the interface. Clinically, this means that material selection and fabrication technique should be judged not only by nominal material properties, cost, or esthetics, but also by their effect on both marginal and internal adaptation, as these factors may influence mechanical stability, sealing capacity, and peri-implant tissue health over time [15, 25].

This study has several limitations. First, it was performed in vitro and therefore cannot fully reproduce the biological, microbial, and functional complexity of the oral environment. Second, the sample size was limited, which is consistent with the pilot nature of the study but reduces statistical power and limits generalizability. In addition, the study did not use a fully crossed material-by-fabrication design; therefore, the independent contribution of material to internal fit could not be determined. Third, micro-CT analysis is inherently dependent on system resolution and reconstruction conditions. In the present study, the maximum spatial resolution was 6.7 μm; therefore, smaller gaps could not be detected. This point may also explain why some groups showed 0.00 μm values in region 1, which should be interpreted as the absence of a detectable gap within the resolution threshold rather than the absolute absence of discrepancy. In addition, high-density materials may generate beam-hardening and streak artifacts that can affect grayscale transitions at the interface despite corrective procedures [9]. More broadly, micro-CT is a valuable nondestructive imaging modality, but its findings depend on scanning parameters, reconstruction and segmentation strategies, and selected outcome measures [38, 45]. Because the present study obtained discrete linear gap measurements at predefined locations, it did not quantify the total interfacial gap volume, contacting-surface percentage, or spatial gap distribution across the entire connection and should not be interpreted as a comprehensive three-dimensional assessment of implant–abutment fit. Future studies should include larger samples, additional materials and connection designs, and volumetric, surface-based, or spatial gap-distribution analyses combined with evaluations of external marginal adaptation, internal fit, and microleakage to better characterize the interface under functional conditions [9, 39, 40].

## Conclusions

Within the limitations of this in vitro study, laser-sintered cobalt–chromium abutments showed less favorable internal adaptation than the milled and cast cobalt–chromium groups. These findings suggest that the manufacturing workflow and quality-control verification should be considered when precise seating at the implant–abutment connection is required, particularly for laser-sintered components. However, because validated clinical thresholds for micro-CT-derived internal gap measurements are unavailable and the observed differences were not directly associated with mechanical or biological complications, their clinical significance cannot be established. Therefore, these findings should not be used to independently determine the selection or exclusion of a fabrication workflow. Clinical studies correlating gap magnitude and distribution with micromovement, mechanical complications, microleakage, and peri-implant tissue outcomes are needed. 

## Data Availability

No datasets were generated or analysed during the current study.
